# Patterns of failure after use of ^18^F-FDG PET/CT in integration of extended-field chemo-IMRT and 3D-brachytherapy plannings for advanced cervical cancers with extensive lymph node metastases

**DOI:** 10.1186/s12885-016-2226-0

**Published:** 2016-03-03

**Authors:** Yih-Lin Chung, Cheng-Fang Horng, Pei-Ing Lee, Fong-Lin Chen

**Affiliations:** Department of Radiation Oncology, Koo Foundation Sun Yat-Sen Cancer Center, No.125 Lih-Der Road, Pei-Tou district, Taipei, 112 Taiwan; Department of Medical Research, Koo Foundation Sun Yat-Sen Cancer Center, Taipei, Taiwan; Department of Nuclear Medicine, Koo Foundation Sun Yat-Sen Cancer Center, Taipei, Taiwan; Department of Medical Physics, Koo Foundation Sun Yat-Sen Cancer Center, Taipei, Taiwan

**Keywords:** Cervical cancer, ^18^F-FDG PET/CT, IMRT, Brachytherapy, Pattern of failure, Disease-free survival

## Abstract

**Background:**

The study is to evaluate the patterns of failure, toxicities and long-term outcomes of aggressive treatment using ^18^F-FDG PET/CT-guided chemoradiation plannings for advanced cervical cancer with extensive nodal extent that has been regarded as a systemic disease.

**Methods:**

We retrospectively reviewed 72 consecutive patients with ^18^F-FDG PET/CT-detected widespread pelvic, para-aortic and/or supraclavicular lymph nodes treated with curative-intent PET-guided cisplatin-based extended-field dose-escalating intensity-modulated radiotherapy (IMRT) and adaptive high-dose-rate intracavitary 3D-brachytherapy between 2002 and 2010. The failure sites were specifically localized by comparing recurrences on fusion of post-therapy recurrent ^18^F-FDG PET/CT scans to the initial PET-guided radiation plannings for IMRT and brachytherapy.

**Results:**

The median follow-up time for the 72 patients was 66 months (range, 3–142 months). The 5-year disease-free survival rate calculated by the Kaplan-Meier method for the patients with extensive N1 disease with the uppermost PET-positive pelvic-only nodes (26 patients), and the patients with M1 disease with the uppermost PET-positive para-aortic (31 patients) or supraclavicular (15 patients) nodes was 78.5 %, and 41.8–50 %, respectively (N1 vs. M1, *p* = 0.0465). Eight (11.1 %), 18 (25.0 %), and 3 (4.2 %) of the patients developed in-field recurrence, out-of-field and/or distant metastasis, and combined failure, respectively. The 6 (8.3 %) local failures around the uterine cervix were all at the junction between IMRT and brachytherapy in the parametrium. The rate of late grade 3/4 bladder and bowel toxicities was 4.2 and 9.7 %, respectively. When compared to conventional pelvic chemoradiation/2D-brachytherapy during 1990–2001, the adoption of ^18^F-FDG PET-guided extended-field dose-escalating chemoradiation plannings in IMRT and 3D-brachytherapy after 2002 appeared to provide higher disease-free and overall survival rates with acceptable toxicities in advanced cervical cancer patients.

**Conclusions:**

For AJCC stage M1 cervical cancer with supraclavicular lymph node metastases, curability can be achieved in the era of PET and chemo-IMRT. However, the main pattern of failure is still out-of-field and/or distant metastasis. In addition to improving systemic treatment, how to optimize and integrate the junctional doses between IMRT and 3D-brachytherapy in PET-guided plannings to further decrease local recurrence warrants investigation.

**Electronic supplementary material:**

The online version of this article (doi:10.1186/s12885-016-2226-0) contains supplementary material, which is available to authorized users.

## Background

Advanced cervical cancer requires multimodal treatment. Because of the high probabilities of pelvic, para-aortic and occult supraclavicular lymph node metastasis that is part of the TNM staging system but not part of the International Federation of Gynecology and Obsterics (FIGO) staging system, pre-treatment lymph node staging by using [18 F]fluorodeoxyglucose positron emission tomography/computed tomography (^18^F-FDG PET/CT) to detect potential disease that might have been missed by conventional imaging has been recommended [1]. A series of studies demonstrated that at diagnosis, up to 47 % of cervical patients had lymph node metastasis on PET [2].

The presence of PET-positive lymph nodes may identify patients who are better treated with cisplatin-based concurrent chemoradiotherapy (CCRT) to minimize the risk of increased toxicities associated with a combination of surgery and radiotherapy (RT) [3, 4]. However, conventional four-field box or anterior-posterior parallel opposed RT for patients with extensive multiple pelvic/para-aortic/supraclavicular lymph node metastases is difficult to escalate dosage to the para-aortic and bulky sidewall nodes owing to the risk of severe complications such as enteritis, proctitis and cystitis [5]. Since the adoption of new RT modalities (eg, intensity-modulated RT (IMRT), image-guided IMRT (IGRT) and three-dimension (3D)-brachytherapy results in fewer treatment-related normal tissue toxicities, dose escalation might improve local control and even survival by employing extended-field IMRT/IGRT CCRT that aggressively targets the lymph node regions according to the highest level of lymph node involvement detected by PET [6–9].

However, there are yet no trials that compare curative-intent extended-field CCRT (to cover from the pelvic, para-aortic to supraclavicular fossa) versus palliative-intent pelvic-only CCRT. It remains unknown whether the PET-based treatment guideline regarding radical hysterectomy versus definitive CCRT and PET-guided IMRT/IGRT/brachytherapy planning to increase tumor coverage and treatment intensity improves survival, or simply induces the phenomenon of “TNM stage migration” and “treatment selection bias” in cervical cancer [10].

In this study, we assessed the long-term outcomes, patterns of failures and toxicities in advanced cervical cancer patients with extensive FDG-avid pelvic, para-aortic, and/or supraclavicular metastases but no known bone and/or visceral disease at diagnosis. They were all treated with curative-intent extended-field dose-escalating CCRT by IMRT/IGRT/3D-brachytherapy targeting all PET-positive lymph node basins and boosting lesions with standardized uptake values (SUVs) of 2.5 or greater. We also compared the survival outcomes of invasive cervical cancer before and after 2002, when ^18^F-FDG PET/CT was set up for cancer staging and PET-guided IMRT, IGRT and 3D-brachytherapy plannings became standard and common practice at our institution with time.

## Methods

### Patients

This study was approved by the ethics committee of Koo Foundation Sun-Yat-Sen Cancer Center. We retrospectively reviewed 564 consecutive biopsy-proven cervical cancer patients with FIGO stage IA2-IVA or IVB that had para-aortic and/or supraclavicular lymph node involvement with no known bone and/or visceral metastasis at diagnosis between 1990 and 2010. Written informed consent was obtained from all patients included in the study before therapy. This study was performed in accordance with the Declaration of Helsinki and with national regulations.

### Staging

After 2002, patients with bulky IB2, FIGO IIB or higher stage, or magnetic resonance imaging (MRI)-positive pelvic lymphadenopathy further underwent ^18^F-FDG PET/CT to detect occult extrapelvic metastasis (Additional file 1: Fig. S1). The extrapelvic foci of increased FDG uptake on PET were always confirmed by CT- or sonography-guided or laparoscopic biopsy and/or cytology. Although the nodal status was determined by MRI and PET images and even surgical procedures, results of the MRI- and PET-based AJCC TNM staging did not alter the initial clinical FIGO stage. However, treatment strategy and planning were based on the PET- and MRI-findings.

### Curative treatment

Treatment options for early stage patients with FIGO stage IA2-IIA disease included primary surgery as follows: modified radical hysterectomy (class II) and pelvic lymphadenectomy for IA2; radical hysterectomy (class III) and pelvic lymphadenectomy for IB1-IIA without oophorectomy for squamous cell carcinoma, or with oophorectomy for adenocarcinoma; or primary RT without concurrent chemotherapy for IA2-IB1, or with concurrent chemotherapy for IB2-IIA. For patients treated with a primary surgical approach, post-operative adjuvant RT was administered if the final pathology findings revealed intermediate-risk features of lymphovascular invasion or deep stromal invasion; adjuvant CCRT was administered if high-risk features of positive surgical margins, pathologically involved pelvic nodes, or positive parametrial involvement were observed (Additional file 7: Fig. S6A). For advanced stage patients with FIGO IIB-IVA or IVB with para-aortic or supraclavicular lymph node involvement but no distant organ metastasis, definitive CCRT was the mainstay of treatment.

### Extended-field dose-escalating CCRT

In order to overcome the challenges of intra- and inter-fraction organ motion, anatomy variations due to tumor shrinkage, and target dose escalation while sparing normal tissues during a long course of fractionated RT, the RT planning combined the advantages of conventional external beam 3D-RT, modern IMRT/IGRT and 3D-brachytherapy techniques to comprise a 3-phase sequential external beam radiation intervening with adaptive 3D-brachytherapy (Additional file 1: Fig. S1, Additional file 2: Fig. S2, Additional file 3: Fig. S3, Additional file 4: Fig. S4, Additional file 5: Fig. S5). Image-guidance and adaptive RT with repeated CT simulation were commonly used together. During dose escalation by IGRT, daily cone-beam CT was used to not only guide re-position by simple couch shifts but also decide to make a new adaptive plan to prevent suboptimal treatment.

Advanced cervical cancer patients all received extended-field 3D-RT (10 or 18 MV photons, 1.8 Gy per fraction, 1 fraction per day, 5 fractions per week) from the pelvis to the para-aortic area, depending on their work-up, with concurrent weekly cisplatin (40 mg/m^2^) for 6 cycles. For patients with chronic renal failure or severe baseline neuropathy which could not be improved by a ureteral stent or nephrostomy tube placement, we treated these patients with weekly carboplatin dosed at area under the curve (AUC) 2 for 6 cycles. All patients underwent a pre-treatment computed tomography (CT)-based simulation with a full bladder and an empty rectum. Delineation of the cervical tumor, enlarged lymph nodes, uterus, bladder, rectum, intestine, femurs, and kidneys was based on dosimetric CT scans acquired with axial 3–5 mm thickness. For patients with extensive lymph node involvement, the PET scans and the RT simulation CT images were fused using point and anatomic matching to allow contouring all of the metabolically active lymph nodes with SUVs of 2.5 or greater at the delayed phase. A 0.5-cm to 1.0-cm margin was added to the PET-detected or gross nodes to create the clinical target volume (CTV). An extra 0.5-cm to 1-cm was added to CTV to form a planning target volume (PTV). Patients underwent an additional CT simulation for adaptive IMRT/IGRT re-planning after 4140–4500 cGy. For IMRT planning, the lateral boundary of parametrial CTV was at the pelvic side wall and the medial boundary of parametrial CTV abutted the uterus, cervix and vagina though the superior and inferior boundaries might vary (Additional file 3: Fig. S3). IMRT boost was used after 4500 cGy to treat PTV covering the para-aortic nodes, pelvis and parametria up to 5400 cGy in 30 fractions while sparing the intestine, kidneys, spinal cord, bladder, rectum, and femoral neck. IGRT was used after 5400 cGy to boost CTV covering the para-aortic and pelvic nodes with ^18^F-FDG SUVs of 4.5 or greater at the delayed phase of PET up to 5940–6480 cGy in 33–36 fractions. Chemotherapy was withheld when the white blood cell count was <1500/mL or the platelet count was <80,000/mL, and restarted after recovery from such low cell counts. Ninety percent of patients with definitive CCRT received at least four cycles of cisplatin or carboplatin.

For confluent bulky supraclavicular lymph node metastasis with pathology confirmation (Additional file 5: Fig. S5), a second set of PET-guided RT treatment planning included irradiation of the bilateral lower neck, supraclavicular fossa and upper mediastinum by antero-posterior opposed parallel portals (up to 4500 cGy in 25 fractions over 5 weeks), and then an IMRT boost for CTV covering the PET-detected supraclavicular/mediastinum nodes with SUVs of 4.5 or greater at the delayed phase up to 5940–6120 cGy in 33–34 fractions with sparing of the heart, esophagus, and spinal cord. For the occult supraclavicular metastasis (Additional file 1: Fig. S1), the RT field would not include the upper mediastinum. For patients with good performance status and no anemia or body weight loss, the supraclavicular metastasis was irradiated at the same time with para-aortic/pelvic RT (Additional file 5: Fig. S5); otherwise, it would be irradiated sequentially.

### Adaptive image-based high-dose-rate intracavitary 3D-brachytherapy

After 4500 cGy external beam irradiation, adaptive high-dose-rate PET-guided intracavitary 3D-brachytherapy using an iridium-192 source and Henschke afterloading applicators was performed once or twice weekly under general anesthesia. Patients underwent a pelvic CT scan acquired with axial 1 mm thickness right after implantation. The images were then transferred onto Oncentra^®^ platforms (Nucletron Medical Systems, an Elekta company, Stockholm, Sweden). The PET scans plus MRI T2WI taken before treatment and the post-implantated CT images were fused using anatomic matching. The ICRU-38 point-A, the initial tumor extent and the entire uterine cervix were contoured and summed together as CTV. The CTV was further defined as high risk (HR)- and intermediate risk (IR)-CTVs based on relatively different intensity of SUVs of ^18^F-FDG PET (HR defined as SUVs of 4.5 greater at the delayed phase and IR defined as SUVs of 2.5–4.5 at the delayed phase). Organs at risk (rectum, sigmoid and bladder) and ICRU bladder and rectum points were also contoured. The dose prescription was adjusted to deliver at least 5–7 Gy per fraction using 4–6 insertions to cover both point-A and 90–100 % of the PET-based HR/IR-CTV, based on the dose limit derived from the simulated 3D computer treatment plan for the rectum, sigmoid and bladder, using both the ICRU-38 guidelines and the recommendations of the GEC–ESTRO [11, 12].

### Combination of external beam radiation and brachytherapy

By applying the linear quadratic model to transform brachytherapy and external beam absolute doses to a 2-Gy equivalent dose (EQD_2,_), we could sum the total EQD2 and generate dose volume histograms of CTV and organs at risk. In our protocol, the 1^st^ brachytherapy was performed just after completion of the initial 3D-RT and before the start of IMRT boost so that we could fuse the simulated CT scans of the 3D-RT, IMRT and brachytherapy plannings in the middle of RT course for final total dose adjustment based on the dose constraints to organs at risk. In order to achieve the ESTRO recommended CTV levels and dose constraints for organs at risk, we repeatedly adjusted the parameters of dose constraints for organs at risk in IMRT plannings, and manually reiterated brachytherapy dose-optimization to combine external beam radiation with brachytherapy. Our goal was to deliver at least total 60 Gy EQD2 (α/β = 10) to 90 % of the IR-CTV and a minimum of total 85 Gy EQD2 (α/β = 10) to 90 % of the HR-CTV while limiting organs at risk to a minimal dose of 75 Gy EQD2 (α/β = 3) to the maximally exposed 2 cm^3^ of the rectal wall and of the sigmoid wall, and 85 Gy EQD2 (α/β = 3) to the 2 cm^3^ of the bladder wall (Additional file 1: Fig. S1, Additional file 2: Fig. S2, Additional file 3: Fig. S3, Additional file 4: Fig. S4, Additional file 5: Fig. S5).

### Follow-up

Patients had regular follow-up of physical examinations, Pap tests, and tumor markers (SCC and CEA) approximately every 2–3 months for the first 12 months, every 3–4 months for the following 2 years, every 4–6 months for the next 2 years, and then yearly. A repeat pelvic MRI and/or abdominal/chest CT scans were performed 2 months after completing treatment to evaluate responses, and then annually. A repeat ^18^F-FDG PET/CT was performed when warranted by MRI, tumor markers, clinical examination or symptoms. The sites and timing of any recurrence were recorded. NCI Common Terminology Criteria for Adverse Events (CTCAE v3.0) was used to score the maximum toxicity.

### Statistical analysis

Survival rates were measured from the beginning of treatment, and calculated using the Kaplan-Meier method. The test of equivalence of estimates of overall survival or disease-free survival between the periods 1990–2001 and 2002–2010 was performed using the log-rank test. A value of *p* < 0.05 was set as the threshold for significance.

## Results

### Impacts of ^18^F-FDG PET/CT-guided radiation planning on patterns of failure

We analyzed the treatment results of 72 consecutive advanced cervical cancer patients with extensive pelvic (more than two or bilateral pelvic lymph node metastases), para-aortic, and/or supraclavicular nodes with no known bone and/or visceral metastasis at diagnosis, who were all staged by clinical FIGO system, pelvic MRI and whole body ^18^F-FDG PET/CT scans between 2002 and 2010 (Table 1 and Additional file 1: Fig. S1). FIGO stage II (62.5 %), PET-based AJCC stage M1 (para-aortic and/or supraclavicular lymph node involvement) (63.9 %), and squamous cell carcinoma (86.8 %) were the most common clinical stages and pathology. They were all treated by PET-guided cisplatin-based extended-field dose-escalating external beam radiation and adaptive 3D-brachytherapy with curative intent (Additional file 1: Fig. S1, Additional file 2: Fig. S2, Additional file 3: Fig. S3, Additional file 4: Fig. S4, Additional file 5: Fig. S5).

**Table 1:** Clinical characteristics and FIGO stage distribution of 72 cervical cancer patients with extensive PET-positive pelvic, para-aortic, and/or supraclavicula node disease treated with curative-intent PET-guided extended-field chemo-IMRT/3D-brachytherapy

PET-based staging	N1 (multiple pelvic-only nodes)	M1 (para-aortic and/or supraclavicular nodes without visceral metastasis)	
The PET-detected highest level of lymph node involvement	pelvic	para-aortic	supraclavicular
No. of Patients	26	31	15
Age at diagnosis, years			
Median	50.9	53.1	52.5
Range	32.3–73.6	29.8–73.8	36.9–68.7
Tumor histology (%)			
Squamous cell carcinoma	26 (100)	28 (90.3)	12 (80.0)
Adenocarcinoma	0 (0)	2 (6.5)	3 (20)
Adenosquamous	0 (0)	1 (3.2)	0 (0)
FIGO clinical stage			
IA2-IB2	5 (19.2)	8 (25.8)	4 (26.7)
IIA-IIB	18 (69.2)	18 (58.1)	9 (60.0)
IIIA-IIIB	3 (11.5)	3 (9.7)	1 (6.7)
IVA	0 (0)	2 (6.5)	1 (6.7)

*Abbreviations*: *FIGO* International Federation of Gynecology and Obsterics

**Fig. 1:**
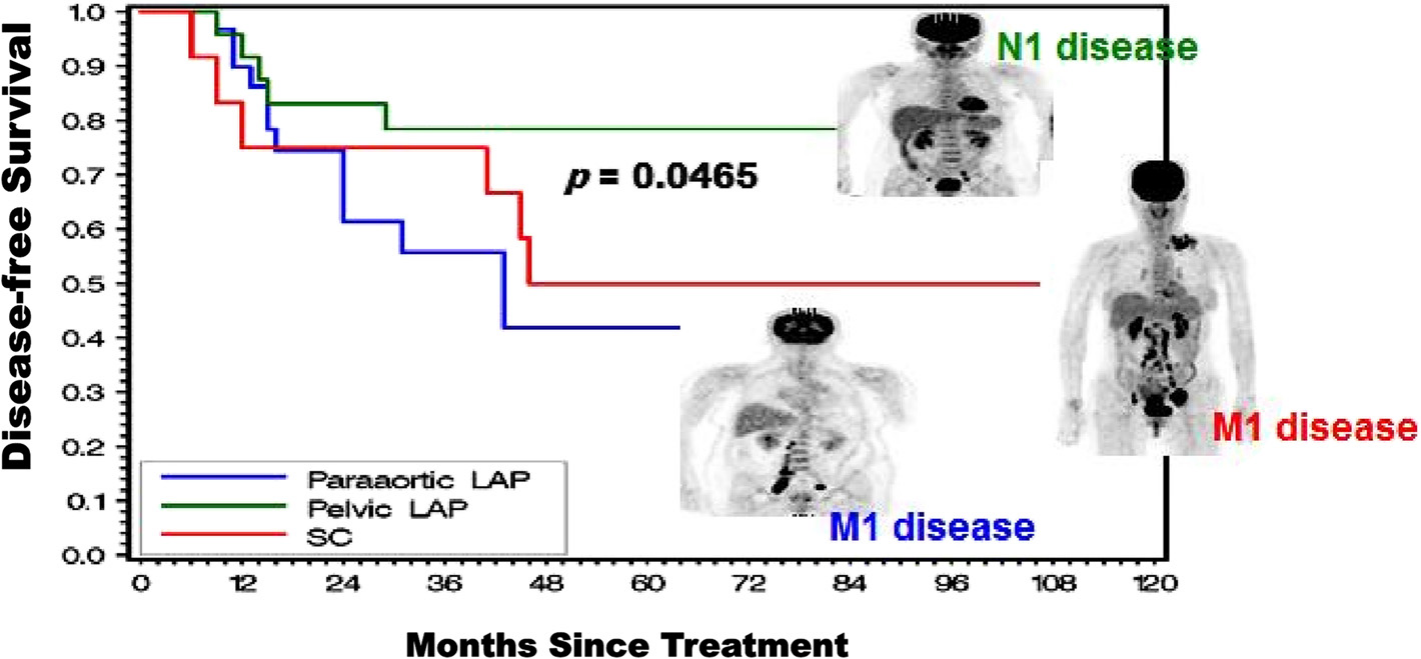
The effects of employing integrated ^18^F-FDG PET/CT staging, modern multi-modalities of radiotherapy (3D-RT, IMRT, IGRT, and 3D-brachytherapy) and concurrent chemotherapy for treatment of advanced cervical cancer with extensive nodal disease but no visceral metastasis at diagnosis. Kaplan-Meier disease-free survival estimates for the 72 patients with extensive PET-positive lymph nodes grouped by their highest level of lymph node involvement after curative-intent treatment. LAP, lymphadenopathy; SC, supraclavicular

The median follow-up time for the 72 patients was 66 months (range, 3–142 months). The 5-year MRI-based disease-free survival or progression-free survival in patients with the uppermost PET-positive pelvic (26 patients), para-aortic (31 patients), and supraclavicular (15 patients) nodes were 78.5, 41.8, and 50 %, respectively (pelvic-only nodal disease (26 patients) vs. para-aortic and/or suparclavicular nodal disease (46 patients), *p* = 0.0465) (Fig. 1 and Table 2). On the other hand, the bone and/or visceral metastasis rates for patients with the uppermost PET-positive pelvic, para-aortic, and supraclavicular nodes were 23.1, 32.3, and 33.3 %, respectively. These findings are consistent with the TNM system, which stages pelvic lymph node metastasis as N1, and para-aortic or supraclavicular lymph node metastasis as M1. Most recurrences developed 1–3 years after treatment.

**Table 2:** Patterns of failure and survival in cervical cancers with pelvic, para-aortic and/or supraclavicular lymph node metastasis treated with PET-guided extended-field dose-escalating chemo-IMRT/3D-brachytherapy

Outcomes	Pelvic LAP	Paraaortic LAP	SC LAP	Total (%)
Dead/Total	5/26	12/31	6/15	23/72 (31.9)
In-field failure-only (cervix, lymph nodes)	2	5	1	8 (11.1)
Out-of-field failure-only (lymph node, bone and/or visceral metastases)	5	8	5	18 (25.0)
Both failures	1	2	0	3 (4.2)
Survival				
1-year DFS (%)	91.7	89.9	75.0	–
3-year DFS (%)	78.5	55.8	75.0	–
5-year DFS (%)	78.5	41.8	50.0	–

*Abbreviations*: *CCRT* concurrent chemoradiotherapy, *IMRT/IGRT* intensity-modulated and image-guided radiotherapy, *LAP* lymphadenopathy, *DFS* disease-free survival, *PET* fluorodeoxyglucose position emission computed tomography

**Fig. 2:**
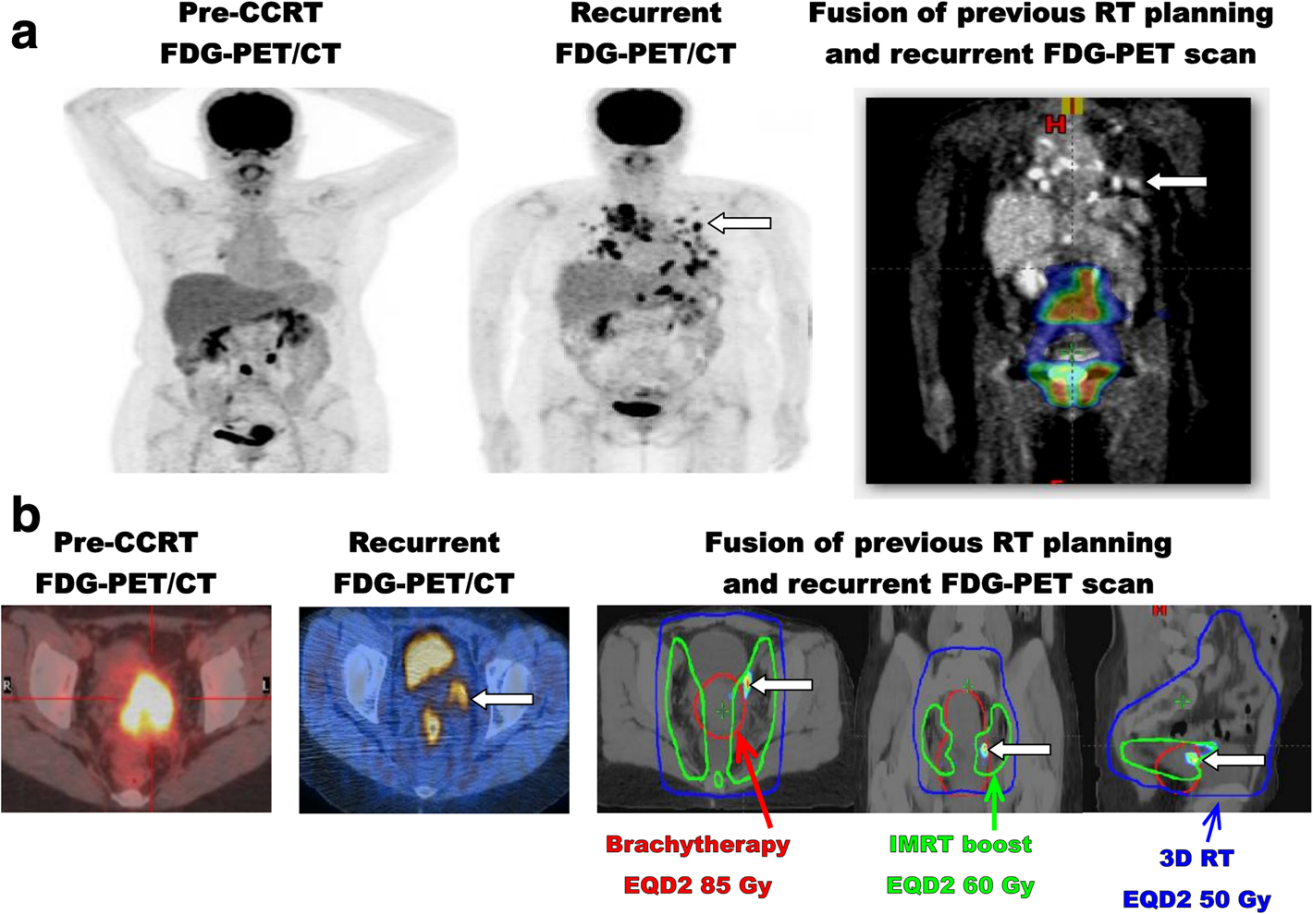
Patterns of failure after ^18^F-FDG PET-guided RT planning. Pre-treatment combined RT planning scans of 3D-RT, IMRT and 3D-brachytherapy are fused to post-treatment recurrent ^18^F-FDG PET/CT scans to map the recurrent tumors in the initial RT treatment fields and dose distribution. The doses of external beam radiation and brachytherapy are transformed to EQD2 (equivalent dose to a 2-Gy fraction) for combination. **a** Out-of field recurrence and distant metastasis. RT dose distribution is demonstrated by colors. The lung metastasis confirmed by pathology is indicated by a white arrow. Note that the post-RT in-field structures show lower metabolic activity as compared to those in the pre-RT scan. (**b**) In-field recurrence. Note that the FDG-avid recurrent cervical tumor (white arrow) confirmed by pathology is located at the junctional zone of IMRT (EQD2 60 Gy) and brachytherapy (EQD2 85 Gy) in the parametrium

In order to assess the patterns of failure, the pre-treatment planning CT scans for PET-guided IMRT and brachytherapy were co-registered and fused to the post-treatment ^18^F-FDG PET/CT scans in patients with recurrence. Recurrent tumors were mapped to the initial RT treatment fields and dose distribution. The main pattern of failure was still out-of-field and/or distant metastasis (N1, 23.1 % vs. M1, 32.6 %) (Fig. 2a). The rate of in-field failure (within 4500–6120 cGy coverage) in the 26 patients (N1) with numerous pelvic-only nodes and the 46 patients (M1) with widespread para-aortic and/or supraclavicular nodes was 11.5 and 17.4 %, respectively. When external beam radiation and intracavitary brachytherapy doses transformed to EQD2 (equivalent dose in 2-Gy per fraction) were combined, we found that the 6 local recurrence around the uterine cervix all fell at the junctional zone between brachytherapy (EQD2 85Gy) and IMRT (EQD2 60 Gy) in the uterosacral and cardinal ligaments or parametrium (Fig. 2b).

### Toxicities

Although the 72 patients completed the curative-intent treatment without major interruption within 56–63 days, almost all of these patients experienced a transient acute grade 2–3 hematologic toxicity with white blood cell count falling to 1000-3000/mm^3^ during the final week of treatment (Additional file 5: Fig. S5B), as well as manageable grade 2 gastrointestinal effects with nausea, vomiting, and/or diarrhea during the treatment course. The late grade 3/4 sequelae were urinary complications in 3 patients (4.2 %) and rectal or bowel complications in 7 patients (9.7 %) (Table 3), suggesting no evidence of excessive severe treatment-related toxicities in our study when compared with the previous reports regarding cervical cancer with pelvic CCRT using a standard RT dosage and technique [13–15].

**Table 3:** Grade 3/4 (CTCAE v3.0) bladder and bowel late complications after PET-guided extended-field dose-escalating chemo-IMRT/3D-brachytherapy

Patient number (%)	Supraclavicular, 15	Paraaortic, 31	Pelvic, 26	Total, 72
Bladder	–	–	–	3 (4.2)
Cystitis	0	1	1	2
Vesicovaginal fistula	0	1	0	1
Bowel	–	–	–	7 (9.7)
Rectal ulcer	1	0	0	1
Proctitis	1	0	0	1
Rectovaginal fistula	1	1	1	3
Bowel obstruction	0	1	1	2

*Abbreviations*: *CTCAE v3.0* common terminology criteria for adverse events, version toxicity, *IMRT/IGRT* intensity-modulated and image-guided radiotherapy, *PET* fluorodeoxyglucose position emission computed tomography

### Improved survival of advanced cervical cancer with time in the era of PET and chemo-IMRT

The year 2002 represents a new era in which our institution started to adopt the PET-guided IMRT and 3D-brachytherapy techniques for advanced cervical cancer patients. Thus, we analyzed whether survival of advanced cervical cancer patients was improved with time after 2002 at our institution.

The analysis included all 564 consecutive patients with newly diagnosed invasive cervical cancer, including FIGO IA2-IVA and IVB without bone/visceral metastasis, at our institution from January 1990 to December 2010 (Additional file 6: Table S1). The two “1990–2001” and “2002–2010” groups featured similar age, histology and stage distribution. The 564 patients were divided into two groups to assess changes in survival outcome between 1990–2001 (229 patients) and 2002–2010 (335 patients). The median follow-up for all patients was 89.9 months (range, 1–249.2 months); the median follow-up was 147.5 months (range, 1–249.2 months) for patients during 1990–2001, and 58.5 months (range, 1–106 months) for patients during 2002–2010.

**Fig. 3:**
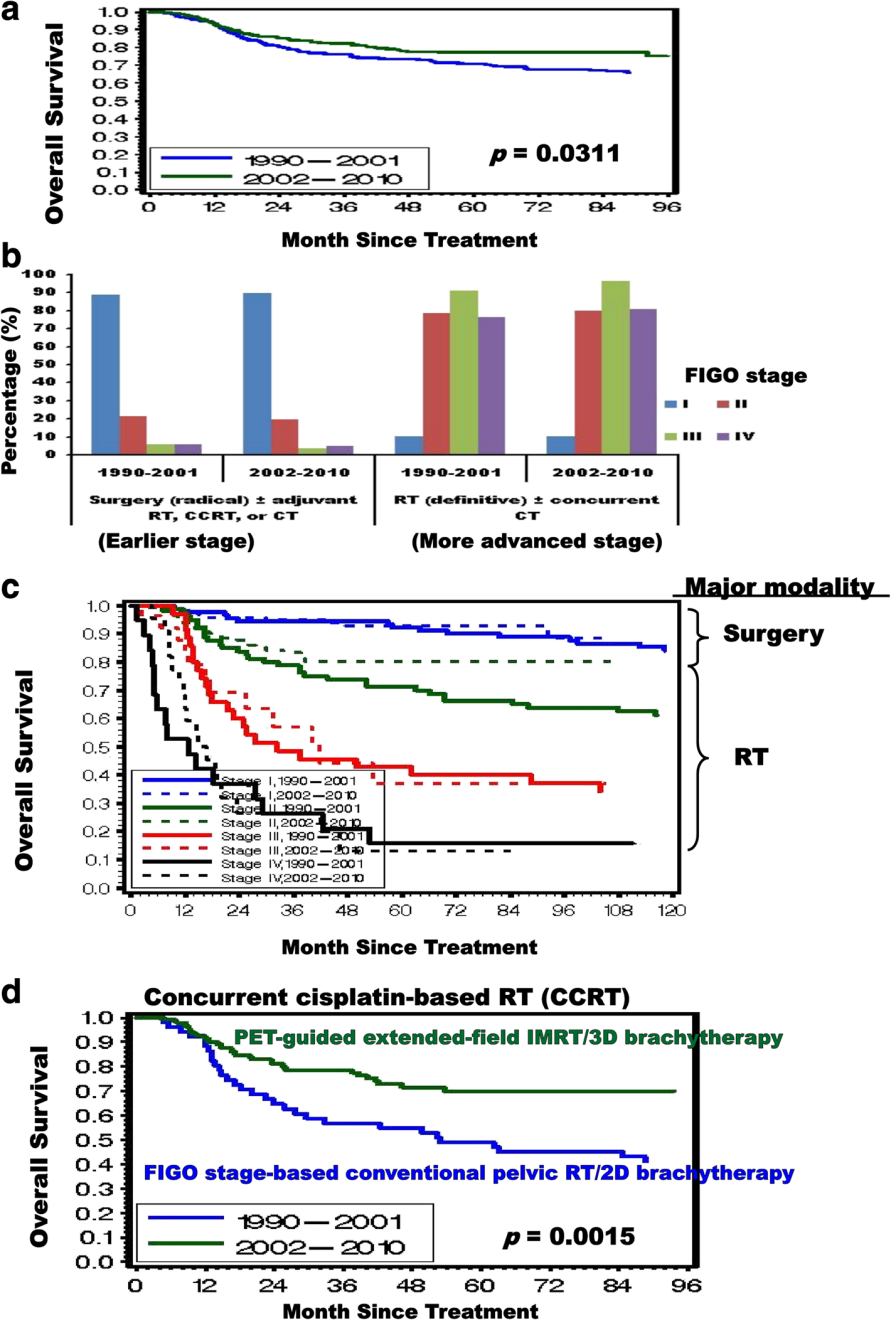
Improved survival of cervical cancer with time in the era of ^18^F-FDG PET/CT and chemo-IMRT/IGRT/3D-brachytherapy: a 20-year analysis including consecutive 564 patients during 1990–2010 in one institution. **a** The overall survival rates for cervical cancer patients (FIGO IA2-IVA, and IVB without visceral metastasis) diagnosed at our institution from 1990 to 2010 are calculated by the Kaplan-Meier method and stratified by treatment year. (**b**) Comparison of the distribution of treatment modalities in each corresponding International Federation of Gynecology and Obstetrics (FIGO) stage, 1990–2001 vs. 2002–2010. RT, radiotherapy; CT, chemotherapy. (**c**) Kaplan-Meier survival estimates for patients with curative treatment are stratified by International Federation of Gynecology and Obstetrics (FIGO) stage and treatment year. (**d**) Kaplan-Meier survival estimates for advanced cervical cancer patients treated with definitive concurrent chemoradiation (CCRT) stratified by treatment year (conventional pelvic CCRT plus 2D brachytherapy in 1990–2001 vs. ^18^F-FDG PET-guided extended-field dose-escalating chemo-IMRT-brachytherapy in 2002–2010)

The 5-year and 8-year overall survival (OS) for patients were 70.7 and 65.9 % in 1990–2001 versus 77.1 and 75.2 % in 2002–2010, respectively (*p* = 0.0311) (Fig. 3a). The assignment of either surgery or RT as a primary modality in corresponding FIGO stages (radical hysterectomy plus lymph node dissection + −adjuvant therapy for FIGO stage IA2-IIA and definitive RT + −chemotherapy for FIGO stages IIB-IVB) were similar or identical between 1990–2001 and 2002–2010 (Fig. 3b). However, the RT technique and dosage were different between 1990–2001 and 2002–2010. We then quantified and compared the relative magnitude of OS improvements in each corresponding FIGO stage between 1990–2001 and 2002–2010 (Fig. 3c). Our results consistently demonstrated that the clinical FIGO I-IV staging was still prognostic after treatment. There was no difference between the two periods regarding survival outcome from 1 to 8 years in FIGO stage I patients for whom surgery was the major treatment. In contrast, for FIGO stage II patients for whom RT was the major modality, the survival rates at 3–8 years were markedly higher in patients treated with para-aortic extended-field dose-escalating modern RT during 2002–2010 than in those treated with conventional pelvic RT during 1990–2001. Thus, our results indicated that the survival improvement after 2002 might not be related to surgery but should be associated with RT. Interestingly, after 2002 the improved OS rate of clinical FIGO II stage patients was very similar to that of PET-based T1-4aN1M0 stage IIIB-IVA disease (Figs. 1 and 3c). The benefit of para-aortic extended-field RT after 2002 was in line with the 10–25 % prevalence of para-aortic lymph node metastasis in locally advanced cervical cancer, which was also consistent with the results of previous studies that demonstrated prophylactic extended-field IMRT with elective para-aortic irradiation improved survival in cervical cancer with PET-positive pelvic lymph nodes and PET-negative para-aortic lymph nodes [16, 17]. However, most of the FIGO stage III and IV patients treated by dose-escalating RT on lesions with high SUVs of PET during 2002–2010 only exhibited delayed disease progression, and just showed a better survival trend within 3 years than the same FIGO stage patients treated during 1990–2002. Nearly one third of the patients with FIGO stage III to IVA in 1990–2001 or 2002–2010 developed distant metastases 1–3 years after treatment.

Because CCRT was a curative modality for advanced cervical cancer (FIGO stage IB2 to IVA) [13–15], in a specific comparison of the CCRT groups between 1990–2001 (FIGO-based conventional pelvic CCRT/2D-brachytherapy) and 2002–2010 (PET-guided extended-field dose-escalating chemo-IMRT/3D-brachytherapy), we found that the 8-year OS rate in advanced cervical cancer patients greatly improved from 41.2 % in 1990–2001 to 70.1 % in 2002–2010 (*p* = 0.0015) (Fig. 3d). However, it is unclear whether the survival benefit found in this retrospective study was due to better ^18^F-FDG PET/CT-staging or more aggressive treatment by modern RT modalities, or both (Additional file 7: Fig. S6).

## Discussion

We used data from our institutional cancer registry during the period from 1990 to 2010 to examine the trend toward improved survival of patients with invasive uterine cervical cancer after curative-intent treatment. We showed that after 2002, patients with advanced cervical cancers experienced OS improvement with the PET-guided extended-field dose escalating IMRT/IGRT CCRT plus adaptive high-dose-rate image-based intracavitary 3D-brachytherapy. The relative magnitude of OS improvement was greatest in patients with FIGO stage II and patients with extensively PET-detected node metastases.

According to historical data, the risks of para-aortic and supraclavicular lymph node metastasis could be up to 21 and 7 %, respectively, for FIGO stage II patients [2]. However, at our institution in 1990–2001, it was difficult to detect occult lymph node metastases without PET, and without IMRT/IGRT even para-aortic and supraclavicular lymph node involvement was often treated with relatively lower RT doses owing to the fear of organ toxicity and incurable potential. The RT dosage for the metastatic pelvic, paraaortic and supraclavicular nodes was escalated to 5940–6480 cGy in 33–36 fractions by IMRT/IGRT in 2002–2010 in contrast to only 5040–5400 cGy in 28–30 fractions by a conventional 4-field Box or 3-D technique used in 1990–2001. Although in our studies the outcomes for patients with PET-positive para-aortic or supraclavicular lymph node metastases after extended-field chemo-IMRT-brachytherapy dose escalation CCRT are still worse than the outcomes for patients with metastatic lymph nodes confined in the pelvis, they are better than outcomes for patients with bone, lung, or liver metastases [18–23], with approximately 40–50 % of patients still living with progression free of disease at 5 years at our institution. Moreover, when compared with historical data showing event-free survival rates at 3 years in cervical patients with PET-detected para-aortic, and with supraclavicular involvement were only 40, and 0 %, respectively, the disease-free survival outcomes at 3 years in our patients with PET-detected M1 disease (para-aortic and/or supraclavicular metastasis) but no known bone and/or visceral metastasis at diagnosis were greatly improved to 55.8–75 % after PET-guided extended-field dose-escalating chemo-IMRT-brachytherapy [2]. The results imply an association between improvement of survival of a subset of AJCC stage M1 cervical cancer patients and advancements in PET staging and modern PET-guided chemo-IMRT-brachytherapy. Thus, the AJCC may need to re-evaluate re-grouping patients with para-aortic and/or supraclavicular disease in the same stage IVB category as patients with distant bone and/or visceral organ diseases.

In addition to the predominant pattern of out-of-field recurrences and distant organ metastases in twenty-one (29.2 %) of the patients with pelvic, para-aortic and/or supraclavicular nodes after PET-guided extended-field dose-escalating chemo-IMRT, there were still six patients (8.3 %) developing recurrence around the uterine cervix despite the advances in PET-guided IMRT/3D-brachytherapy. Failures out of RT fields could represent insensitive ^18^F-FDG PET/CT for tumor detection before treatment and ineffective systemic chemotherapy, whereas in-field failures may imply resistant tumors or insufficient RT dose. Because brachytherapy has a feature of rapid dose fall-off, it is crucial to know if the local recurrences are close to or at the edge of the brachytherapy target volume that is defined by SUVs of ^18^F-FDG PET. Delineation of spatial relationship between recurrent tumor sites, external beam RT fields and brachytherapy dose-gradient margins is challenging. We compared the location of recurrences on post-therapy ^18^F-FDG PET/CT scans to the integrated EQD2 RT dose distribution from the initial external beam RT and brachytherapy planning scans. The findings of almost all local failures within or around the junctional zone between brachytherapy (EQD2 85 Gy) and IMRT (EQD2 60 Gy) in the bulky cervical tumor edge and involved parametrium seem to imply insufficient dose. Thus, for high-risk patients for local recurrence by evaluation of PET-guided IMRT/intracavitary brachytherapy plannings, electively additional interstitial brachytherapy to the risky parametrium may be considered; otherwise, salvage modified radical hysterectomy following CCRT may increase morbidity. However, there is yet no optimization method that integrates IMRT and brachytherapy to match the dose junction to further boost up tumor dosage without concern of increasing normal tissue toxicities. Moreover, because organ motions of the uterus, bladder, and rectum, and changes in target volume during treatment are significant during cervical cancer treatment, deformable image registration may be more feasible and acceptable for assessing cumulative doses to the tumor and organs at risk in the combination of external beam RT and fractionated brachytherapy.

This is a retrospective study, thus suffering from potential biases. Better supportive care, enhanced multi-disciplinary team cooperation, and greater compliance with updated evidence-based cancer treatment guidelines should also be the likely factors that improved treatment outcomes in the modern management of cervical cancer. Our results show that clinical FIGO staging is still prognostic, and treatment strategy and planning should be based on the PET staging system and modern RT techniques to maximize curable potential and minimize toxicities. However, adoption of a new facility always raises the concern of a Will Rogers phenomenon, which refers to the stage-specific improved survival of patients with cancer by reclassifying them into different prognostic groups owing to the recognition of more subtle disease manifestations through new diagnostic modalities [24]. The Will Rogers phenomenon results in stage migration, which could exhibit improved prognosis without affecting actual survival. Thus, prospective clinical trials comparing management with or without extended-field dose-escalating chemo-IMRT-brachytherapy based on ^18^F-FDG PET/CT findings and plannings are warranted.

Because cervical cancer patients with extensive lymph node involvement are still at high risk of distant metastasis even after ^18^F-FDG PET-guided extended-field dose-escalating treatment, we may need to think about not only other molecular imaging-based anatomic plannings but also tumor biology-based systemic approaches, such as targeting the signaling and/or immune checkpoint pathways that affect human papilloma virus-related oncogenesis and cancer progression [25, 26].

## Conclusions

This study covers the integration of radiation therapy into multimodal treatment approaches in advanced cervical cancer with extensive nodal involvement. We show that improved survival of advanced cervical cancer with time at our institution is correlated with adoption of PET-guided extended-field dose escalating chemo-IMRT and 3D-brachytherapy boost. Although clinical FIGO staging is still prognostic, treatment strategy and planning should be based on modern PET imaging staging and radiation techniques to maximize curable potential and minimize toxicities.

Our results indicate that although advanced cervical cancer with extensive nodal extent has been regarded as a systemic disease by AJCC staging, curability with acceptable toxicities for the M1 stage can still be achieved in near 50 % of the patients treated with modern radiation techniques based on ^18^F-FDG PET/CT findings. However, the main pattern of failure was still out-of-field and/or distant metastasis in 30 % of the patients. In addition to improving systemic treatment, how to optimize the dose junction between IMRT and brachytherapy in PET-guided plannings to further decrease local recurrence also warrants investigation.

## Additional files

Additional file 1: Figure S1. Two Parallel Staging Systems for Advanced Cervical Cancer. (DOC 4920 kb) Additional file 2: Figure S2. Flow-chart of curative-intent treatment for advanced cervical cancer patients with extensive nodal disease but no known visceral metastasis in the era of PET and IMRT at our institution. (DOC 71 kb) Additional file 3: Figure S3. PET-guided RT planning for a locally bulky FIGO IB2 cervical cancer with multiple FDG-avid pelvic-only nodes. The relatively different intensity of standardized uptake values (SUVs) of 18 F-FDG PET were used to delineate HR- and IR-clinical target volumes (CTVs) ((high risk defined as SUVs of 4.5 greater at the delayed phase and intermediate risk defined as SUVs of 2.5–4.5 at the delayed phase). A representative of fusion between pre-therapy PET and post-implantated CT scans at time of the 1st brachytherapy after concurrent chemoradiation of 4500 cGy with one tandem and two ovoid applicators inserted into the uterus and vaginal fornix, respectively. The 3D RT, IMRT and brachytherapy doses are transformed to EQD2 (equivalent dose of 2-Gy fraction) for combination. Adding to external beam radiation, the brachytherapy planning aimed to deliver a minimum of total EQD2 85 Gy to 90 % of the HR/IR-CTVs in 5 fractions (Frs). HR-CTV: FDG-avid dark color; IR-CTV: FDG-avid light color; B, FDG-avid bladder. Note that the green, red and yellow arrows indicate the area only receiving external beam radiation, the junction of IMRT and brachytherapy, and the overlapping of IMRT and brachytherapy, respectively. There is yet no optimization method to match the dose junction between IMRT and brachytherapy. (DOC 399 kb) Additional file 4: Figure S4. PET imaging aids in target volume delineation for radiotherapy (RT) planning in a FIGO IIIB cervical cancer patient with extensive lymph node metastases in the pelvic and para-aortic areas. (A) External beam planning. A representative RT treatment plan includes the combination of an initial 4-field box technique (4500 cGy/25 frs) to the whole pelvis and para-aortic area with a subsequent IMRT boost with central pelvic sparing (900 cGy/5 frs) to the PET-detected lymph node basisn and parametria, and a final IGRT boost to the high SUV lymph nodes (720 cGy/4 frs). (B) PET-based intracavitary 3D brachytherapy planning to deliver at least 500 cGy to 90 % of the PET-definied HR-CTV (as SUVs of 4.5 greater at the delayed phase) and IR-CTV (as SUVs of 2.5–4.5 at the delayed phase). The dark yellow arrow indicates a double-J in the right hydroureter. Ure, ureter; B, bladder; R, rectum; Si, sigmoid. (DOC 448 kb) Additional file 5:Figure S5. The integrated 18 F-FDG PET/CT staging, modern multi-modalities of radiotherapy (RT) planning and concurrent chemotherapy for treatment of an advanced cervical cancer patient with extensive pelvic, para-aortic and supraclavicular nodal diseases but no visceral metastasis at diagnosis. (A) An integrated concurrent chemoradiotherapy (CCRT) showing a combination of PET-guided cisplatin-based extended-field dose-escalating IMRT/IGRT and adaptive high-dose-rate (HDR) image-based intracavitary 3D brachytherapy. External beam RT (EBRT) and brachytherapy doses are transformed to EQD2 (equivalent dose of 2-Gy fraction) for combination. HR-CTV: SUVs of 4.5 greater at the delayed phase of PET; IR-CTV: SUVs of 2.5-4.5 at the delayed phase of PET; R, rectum; B, bladder; Si, sigmoid. (B) The hematologic toxicity profile of the patient during the extended-field CCRT. (DOC 258 kb) Additional file 6: Table S1. Clinical characteristics and distribution of treatment modalities of 564 consecutive cervical cancer patients without visceral metastasis diagnosed in1990-2010. (DOC 38 kb) Additional file 7: Figure S6. Clinical impacts of concurrent chemotherapy, IMRT and PET on survival of cervical cancer patients. The year 2002 represents a point at which our institution started to use 18 F-FDG PET/CT for staging, planning and/or follow-up in cancer patients, and commonly adopted the IMRT technique for cancer patients who needed RT. (A) Proportions of patients alive and free of disease at 8 years after treatment with a specific or combined treatment modality during the periods 1990–2001 and 2002–2010.The disease-free survival of patients treated by conventional RT in 1990–2001 was compared to that of patients treated by IMRT in 2002–2010 (conventional RT vs. IMRT). The disease-free survival of patients treated with concurrent chemoradiotherapy (CCRT) was compared to that of patients treated with RT alone (CCRT vs. RT alone). * represents *p* < 0.05 (Chi-squared test). S, surgery; RT, radiotherapy; C, chemotherapy; CCRT, concurrent chemoradiotherapy. (B) Kaplan-Meier survival estimates for invasive cervical cancer patients with or without PET or PET/CT for staging, planning, follow-up, and/or re-staging between 1990 and 2010. Although patients treated in 1990–2001 did not have pre-treatment PET staging, a portion of these patients had PET/CT for follow-up or re-staging when recurrence occurred after 2002. For patients treated in 2002–2010, if MRI did not show significant pelvic (cN1)/para-aortic (cM1) lymphadenopathy, parametrial involvement (cT2b), hydronephrosis (cT3b) and/or bladder/rectal invasion (cT4), no PET/CT was recommended according to our hospital guideline. For patients with recurrence noted after 2002, if PET/CT did not reveal distant metastasis, salvage surgery was performed if feasible. (DOC 51 kb)

## Abbreviations

3D: three-dimension
CCRT: concurrent chemoradiotherapy
CT: computed tomography
CTV: clinical target volume
EQD_2_: equivalent dose in 2-Gy per fraction
F-FDG PET/CT: [18 F]fluorodeoxyglucose positron emission tomography/computed tomography
FIGO: International Federation of Gynecology and Obsterics
HR: high risk
IGRT: image-guided radiotherapy
IMRT: intensity-modulated radiotherapy
IR: intermediate risk
MRI: magnetic resonance imaging
OS: overall survival
PTV: planning target volume
RT: radiotherapy
SUV: standardized uptake value
