# Supplementary material for: Patterns of failure after use of 18F-FDG PET/CT in integration of extended-field chemo-IMRT and 3D-brachytherapy plannings for advanced cervical cancers with extensive lymph node metastases
Source: BMC Cancer. 2016 Mar 3;16:179. doi: 10.1186/s12885-016-2226-0 (PMC4778334; doi:10.1186/s12885-016-2226-0)
Supplement: Additional file 1: Figure S1. — Two Parallel Staging Systems for Advanced Cervical Cancer. (DOC 4920 kb) [file 12885_2016_2226_MOESM1_ESM.doc]

**Additional file 1: Figure S1**

**Two Parallel Staging Systems for Advanced Cervical Cancer**

|  | **Regional lymph nodes (N1)** | **Distant lymph nodes (M1)** |
| --- | --- | --- |
| **FIGO stage** (by physical examination and limited image studies of intravenous pyelogram) | Cannot be assessed | M1 (palpable supraclavicular nodes) |
| **TNM category** (by MRI and PET images, and/or pathological results)  Anatomic site/prognostic grouping  Clinical Staging | N1 (pelvic-only)  Stage IIIB | M1 (including para-aortic or supraclavicular nodes, liver, lung, or bone)  Stage IVB |

Patient #1

FIGO IB1

Patient #2

FIGO IIB

Patient #3

FIGO IIIB

Patient #4

FIGO IVA


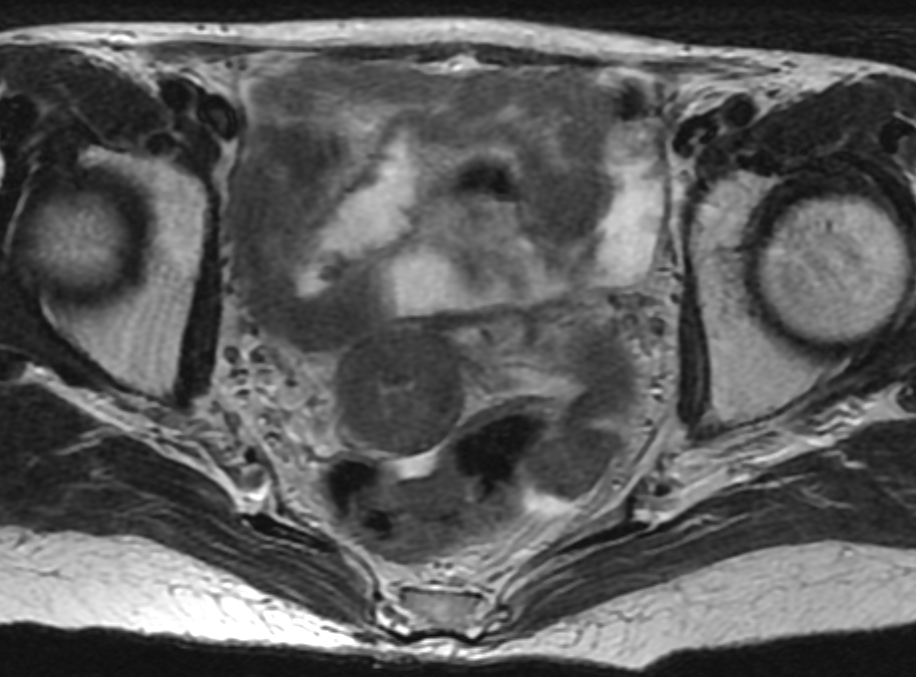

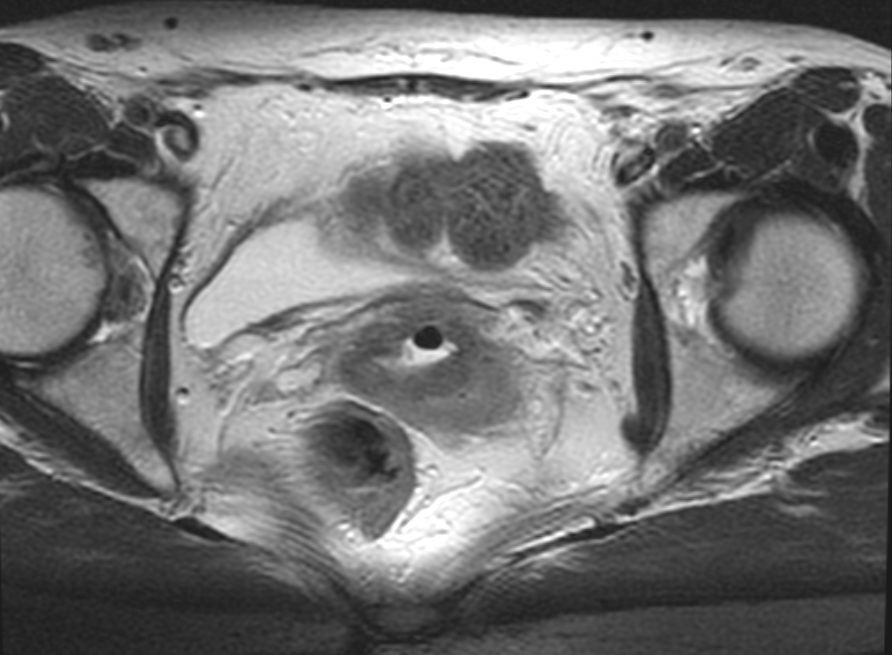

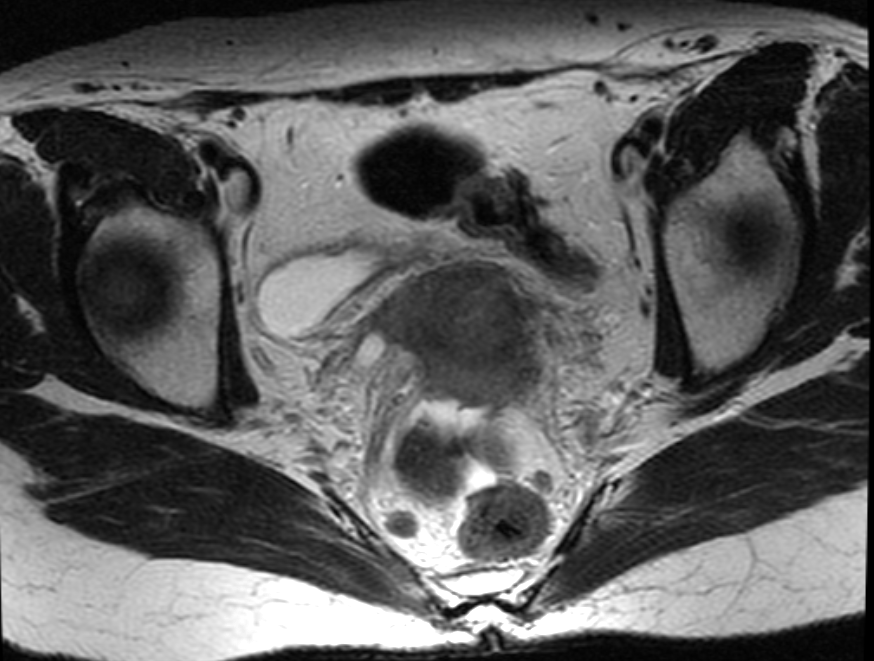

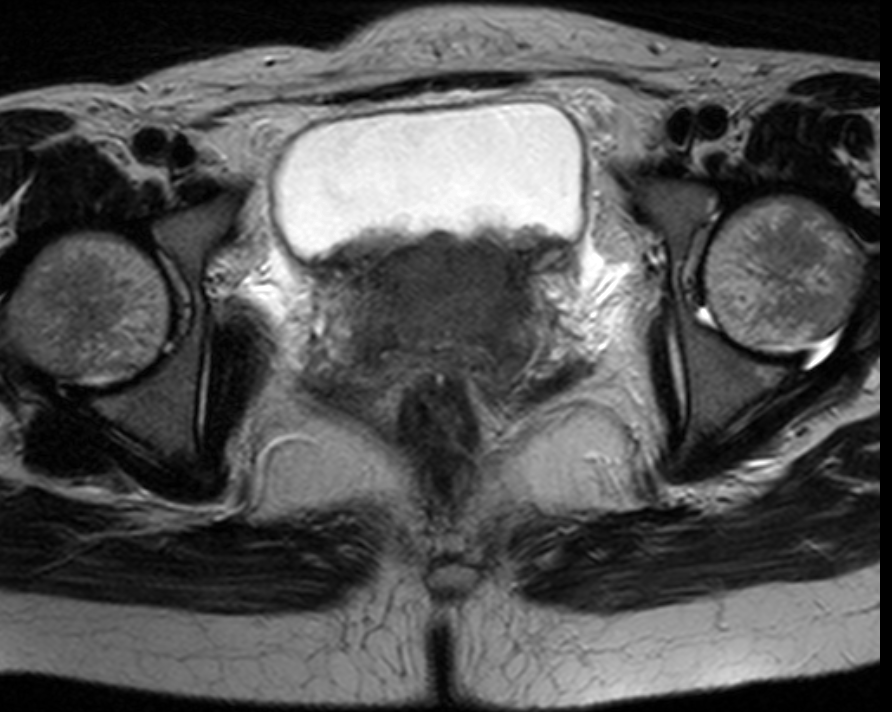


MRI T-staging

MRI N-staging


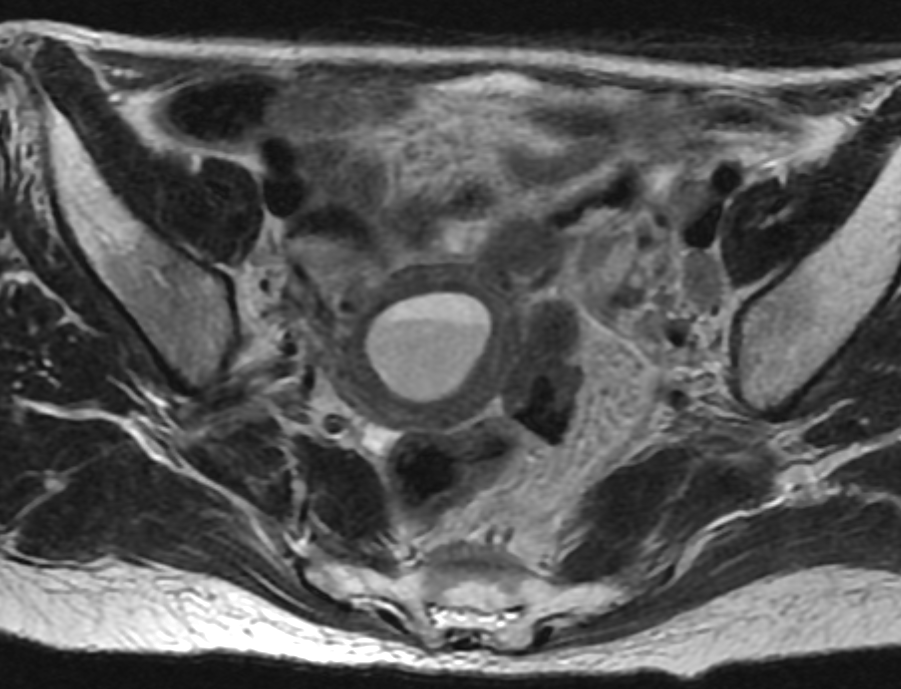

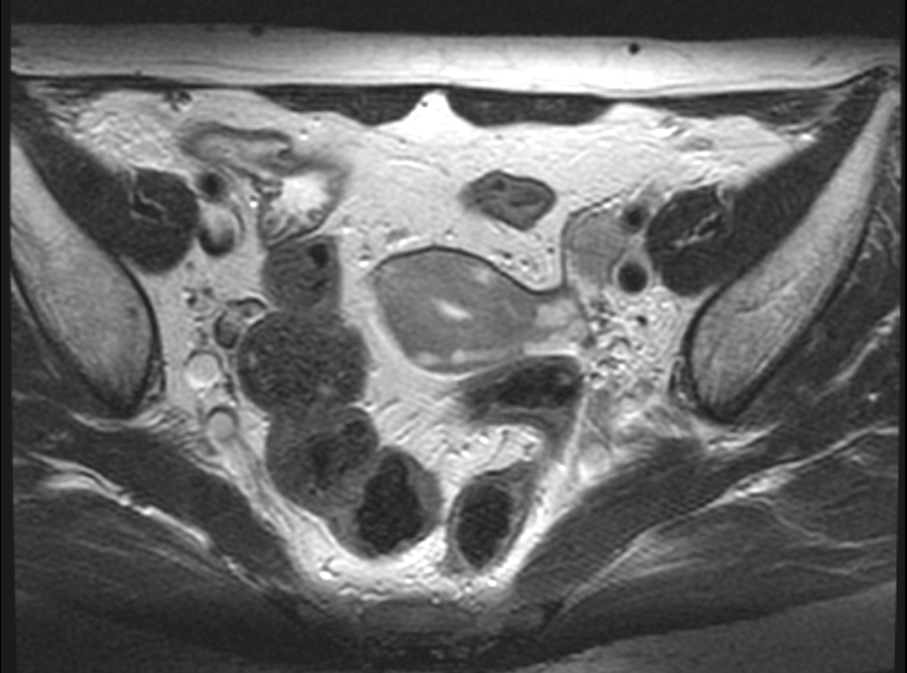

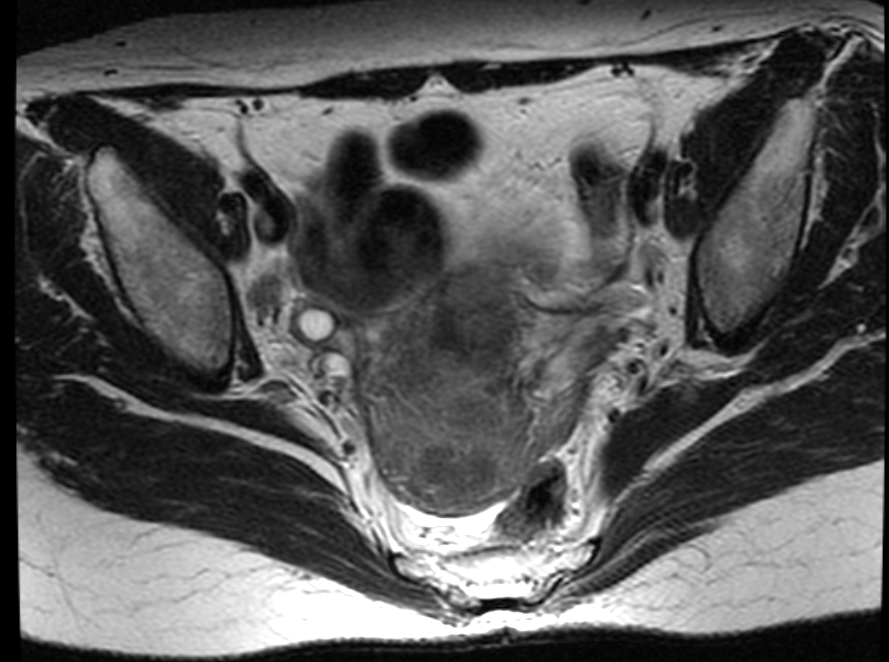

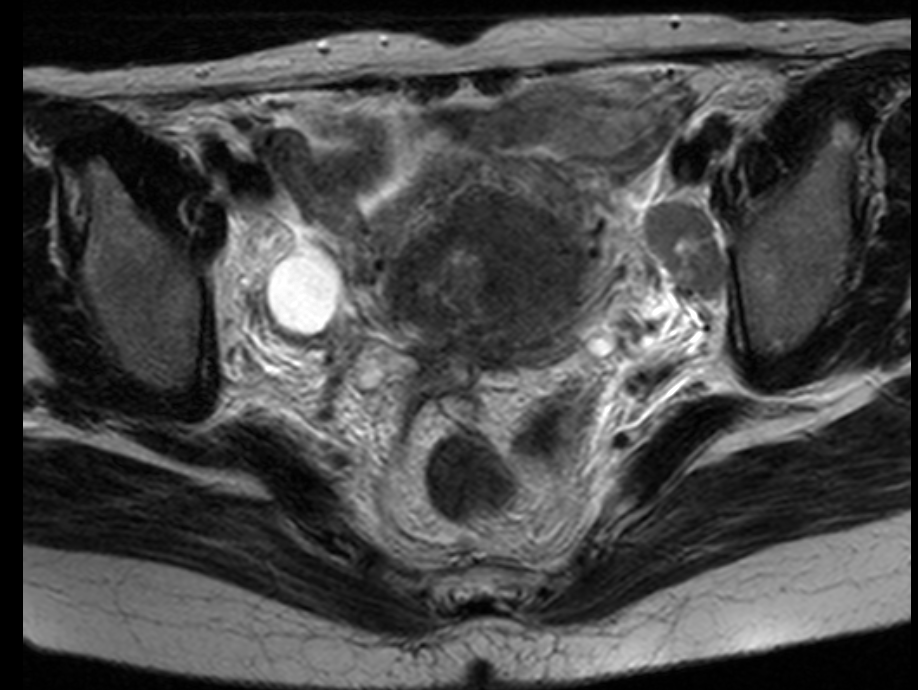


Sonography-guided FNA


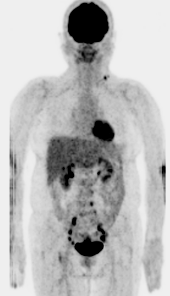

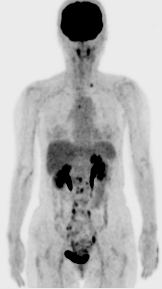

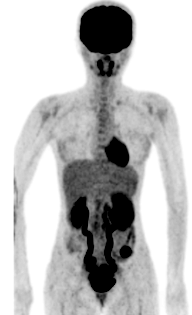

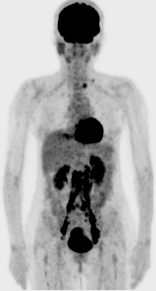


PET-based M-staging


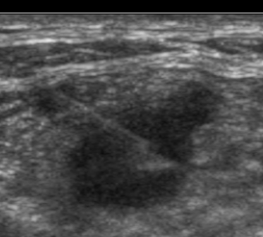


Cytology: Pap staining

Fig. S1. Staging for cervical cancer patients with extensive nodal disease without visceral metastasis. The International Federation of Gynecology and Obstetrics (FIGO) and the American Joint Committee on Cancer (AJCC) are two parallel clinical staging systems for cervical cancer patients. The FIGO staging system mainly for assessing local tumor extent (ie, tumor size, vaginal and parametrial involvement) is based upon physical examination and a limited number of image studies such as intravenous pyelogram, which is difficult to evaluate nodal spread. Although the nodal status can be determined by MRI and PET images and even surgical procedures, results of the MRI- or image-based AJCC TNM evaluation and the PET findings will not alter the initial clinical FIGO stage. Four representatives of the study demonstrate that cervical cancer patientss with different clinical FIGO stages IB1-IVA may have a similar extensive para-aortic node disease and even distant supraclavicular spread, which is staged by the TNM system as M1 disease. However, for such kinds of patients with extensive nodal disease but no known visceral metastasis at diagnosis, the benefit and risk of aggressive treatment for the stage IVB patients with curative intent in the era of PET-guided IMRT remain to be evaluated. The blue arrow indicates the MRI-detected parametrial invasion in a patient with FIGO II stage. The green arrow indicates the MRI-detected hydroureter in a patient with FIGO IIIB. The pink arrow indicates the MRI-detected bladder invasion in a patient of FIGO stage IVA. The orange broken circles indicate the MRI-detected pelvic nodes. The red broken circles indicate the PET-detected occult supraclavicular node spread, which has been confirmed by cytology via sonography-guided fine-needle aspiration.
