# Supplementary material for: Patterns of failure after use of 18F-FDG PET/CT in integration of extended-field chemo-IMRT and 3D-brachytherapy plannings for advanced cervical cancers with extensive lymph node metastases
Source: BMC Cancer. 2016 Mar 3;16:179. doi: 10.1186/s12885-016-2226-0 (PMC4778334; doi:10.1186/s12885-016-2226-0)
Supplement: Additional file 2: Figure S2. — Flow-chart of curative-intent treatment for advanced cervical cancer patients with extensive nodal disease but no known visceral metastasis in the era of PET and IMRT at our institution. (DOC 71 kb) [file 12885_2016_2226_MOESM2_ESM.doc]

**Additional file 2: Figure S2**

26 patients

31 patients

15 patients

MRI-based multiple enlarged pelvic nodes

PET-based

**multiple pelvic node positive**;

para-aortic node negative;

supraclavicular node negative;

visceral metastasis negative

PET-based

**multiple pelvic node positive;**

**extensive para-aortic node positive;**

supraclavicular node negative;

visceral metastasis negative

PET-based

**multiple pelvic node positive;**

**extensive para-aortic node positive;**

**occult supraclavicular node positive;**

visceral metastasis negative

1. Prophylactic para-aortic extended-field cisplatin-based 3-D RT (CCRT): 4500 cGy/25 Frs
2. IMRT pelvic node boost: 1440 cGy/8 Frs
3. Brachytherapy to EQD2 85 Gy
4. Definitive para-aortic extended-field cisplatin-based 3-D RT (CCRT): 4500-5040 cGy/25-28 Frs
5. IMRT para-aortic and pelvic boost: 1440 cGy/8 Frs
6. IGRT para-aortic and pelvic node dose escalation: 360-540 cGy/2-3 Frs
7. Brachytherapy to EQD2 90 Gy

Sonography-guided fine-needle aspiration for the PET-detected occult supraclavicular node, if cytology positive,

Two sets of treatment planning:

1. Para-aortic/pelvic

- Para-aortic/pelvic extended-field cisplatin-based 3-D RT (CCRT): 4500-5040 cGy/25-28 Frs
- IMRT para-aortic and pelvic boost: 1440 cGy/8 Frs
- IGRT para-aortic and pelvic node dose: 360-540 cGy/2-3 Frs
- Brachytherapy to EDQ2 90 Gy

1. Mediastinum/supraclavicular/lower neck

- CCRT to the upper mediastinum, bilateral supraclavicular fossa and lower neck: 4500-5040 cGy/25-28 Frs
- IMRT mediastinal/supraclavicular/neck node boost: 1440-1620 cGy/8-9 Frs

**N1 disease**

**M1 disease**

**M1 disease**

Supplementary Figure 2. Flow-chart of curative-intent treatment for advanced cervical cancer patients with extensive nodal disease but no known visceral metastasis in the era of PET and IMRT at our institution.

**(Fig. S3)**

**(Fig. S4)**

**(Fig. S5)**
