# Supplementary material for: Patterns of failure after use of 18F-FDG PET/CT in integration of extended-field chemo-IMRT and 3D-brachytherapy plannings for advanced cervical cancers with extensive lymph node metastases
Source: BMC Cancer. 2016 Mar 3;16:179. doi: 10.1186/s12885-016-2226-0 (PMC4778334; doi:10.1186/s12885-016-2226-0)
Supplement: Additional file 3: Figure S3. — PET-guided RT planning for a locally bulky FIGO IB2 cervical cancer with multiple FDG-avid pelvic-only nodes. The relatively different intensity of standardized uptake values (SUVs) of 18 F-FDG PET were used to delineate HR- and IR-clinical target volumes (CTVs) ((high risk defined as SUVs of 4.5 greater at the delayed phase and intermediate risk defined as SUVs of 2.5–4.5 at the delayed phase). A representative of fusion between pre-therapy PET and post-implantated CT scans at time of the 1st brachytherapy after concurrent chemoradiation of 4500 cGy with one tandem and two ovoid applicators inserted into the uterus and vaginal fornix, respectively. The 3D RT, IMRT and brachytherapy doses are transformed to EQD2 (equivalent dose of 2-Gy fraction) for combination. Adding to external beam radiation, the brachytherapy planning aimed to deliver a minimum of total EQD2 85 Gy to 90 % of the HR/IR-CTVs in 5 fractions (Frs). HR-CTV: FDG-avid dark color; IR-CTV: FDG-avid light color; B, FDG-avid bladder. Note that the green, red and yellow arrows indicate the area only receiving external beam radiation, the junction of IMRT and brachytherapy, and the overlapping of IMRT and brachytherapy, respectively. There is yet no optimization method to match the dose junction between IMRT and brachytherapy. (DOC 399 kb) [file 12885_2016_2226_MOESM3_ESM.doc]

**Additional file 3: Figure S3**

**3D RT 4500 cGy/25 Frs**

**Pelvic nodal and parametrial IMRT boost**

**1440 cGy/8 Frs**

**Brachytherapy x 5 Frs**

**B**

**B**


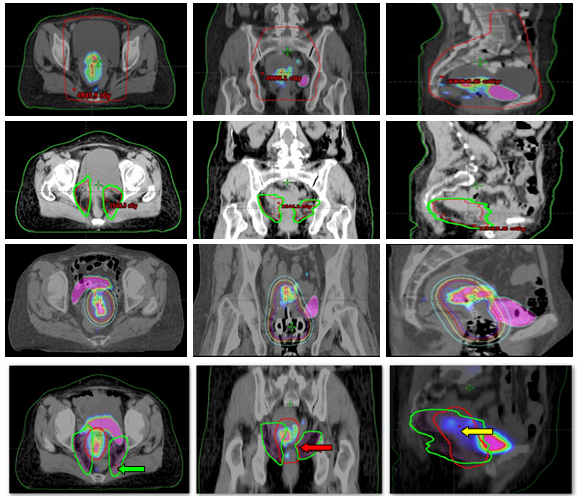
**EQD2 85 Gy: combination of 3D RT, IMRT and brachytherapy**

**Dose summation (EQD2)**

**B**

**B**

**B**

**HR-CTV**

**IR-CTV**

**B**

**B**

**EQD2 58 Gy: combination of 3D RT and IMRT**

Fig. S3. PET-guided RT planning for a locally bulky FIGO IB2 cervical cancer with multiple FDG-avid pelvic-only nodes. The relatively different intensity of standardized uptake values (SUVs) of ^18^F-FDG PET were used to delineate HR- and IR-clinical target volumes (CTVs) ((high risk defined as SUVs of 4.5 greater at the delayed phase and intermediate risk defined as SUVs of 2.5-4.5 at the delayed phase). A representative of fusion between pre-therapy PET and post-implantated CT scans at time of the 1^st^ brachytherapy after concurrent chemoradiation of 4500 cGy with one tandem and two ovoid applicators inserted into the uterus and vaginal fornix, respectively. The 3D RT, IMRT and brachytherapy doses are transformed to EQD2 (equivalent dose of 2-Gy fraction) for combination. Adding to external beam radiation, the brachytherapy planning aimed to deliver a minimum of total EQD2 85 Gy to 90% of the HR/IR-CTVs in 5 fractions (Frs). HR-CTV: FDG-avid dark color; IR-CTV: FDG-avid light color; B, FDG-avid bladder. Note that the green, red and yellow arrows indicate the area only receiving external beam radiation, the junction of IMRT and brachytherapy, and the overlapping of IMRT and brachytherapy, respectively. There is yet no optimization method to match the dose junction between IMRT and brachytherapy.
