# Supplementary material for: Patterns of failure after use of 18F-FDG PET/CT in integration of extended-field chemo-IMRT and 3D-brachytherapy plannings for advanced cervical cancers with extensive lymph node metastases
Source: BMC Cancer. 2016 Mar 3;16:179. doi: 10.1186/s12885-016-2226-0 (PMC4778334; doi:10.1186/s12885-016-2226-0)
Supplement: Additional file 4: Figure S4. — PET imaging aids in target volume delineation for radiotherapy (RT) planning in a FIGO IIIB cervical cancer patient with extensive lymph node metastases in the pelvic and para-aortic areas. (A) External beam planning. A representative RT treatment plan includes the combination of an initial 4-field box technique (4500 cGy/25 frs) to the whole pelvis and para-aortic area with a subsequent IMRT boost with central pelvic sparing (900 cGy/5 frs) to the PET-detected lymph node basisn and parametria, and a final IGRT boost to the high SUV lymph nodes (720 cGy/4 frs). (B) PET-based intracavitary 3D brachytherapy planning to deliver at least 500 cGy to 90 % of the PET-definied HR-CTV (as SUVs of 4.5 greater at the delayed phase) and IR-CTV (as SUVs of 2.5–4.5 at the delayed phase). The dark yellow arrow indicates a double-J in the right hydroureter. Ure, ureter; B, bladder; R, rectum; Si, sigmoid. (DOC 448 kb) [file 12885_2016_2226_MOESM4_ESM.doc]

**Additional file 4: Figure S4**

A


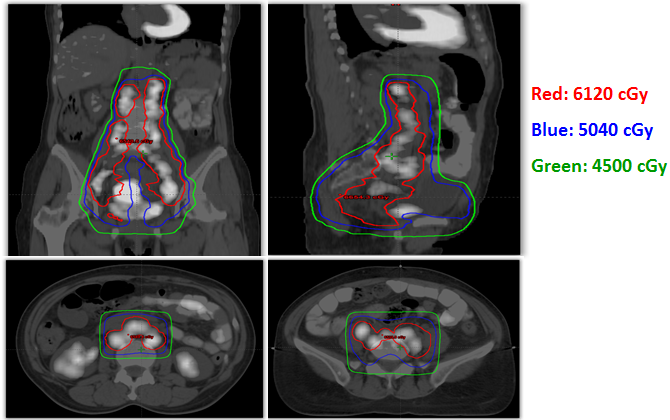


**Coronal view**

**Sagittal view**

**Axial view (paraaortic)**

**Axial view (pelvic)**

RT dose distribution

B


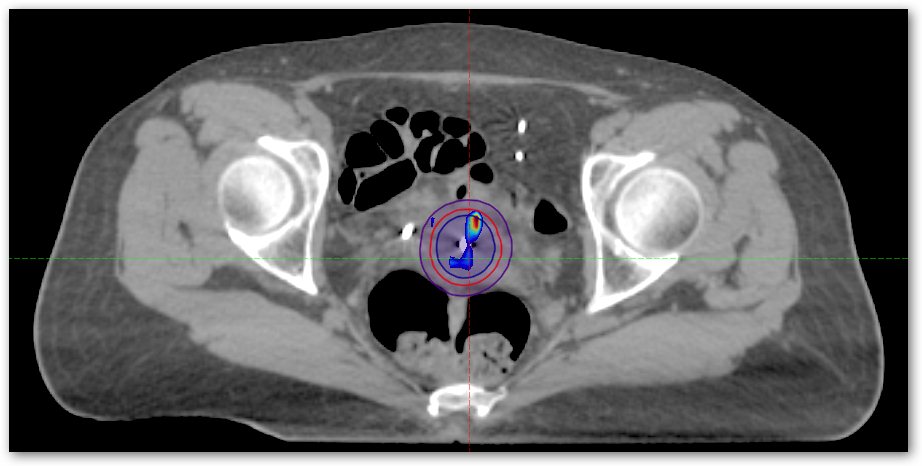

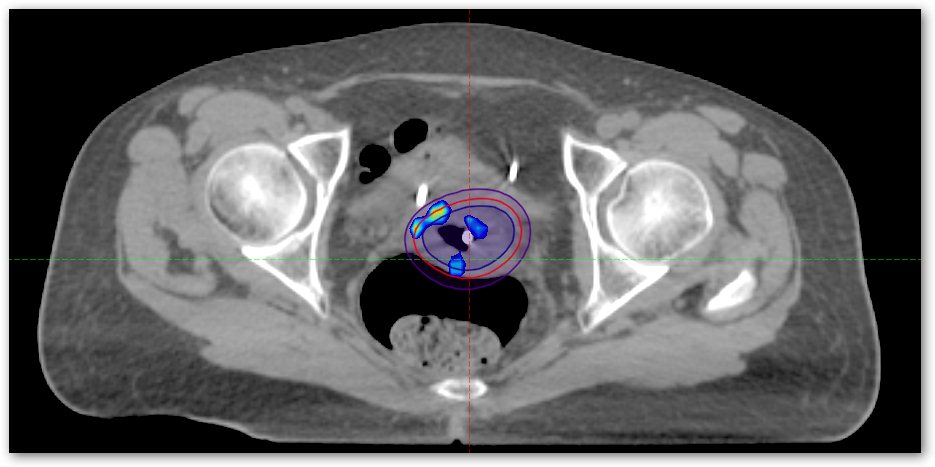


**Axial**

**Axial**

R

R


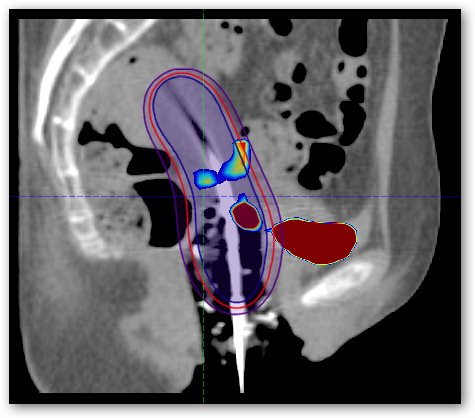

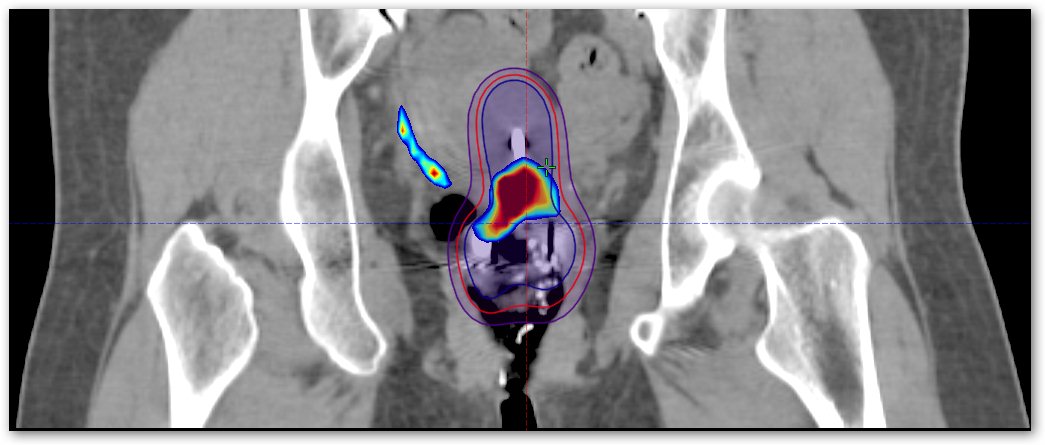


**Sagittal**

R

Si

B

Ure

**Coronal**

**IR-CTV**

**HR-CTV**

Brachytherapy dose gradient 600 cGy 500 cGy 400 cGy

Fig. S4. PET imaging aids in target volume delineation for radiotherapy (RT) planning in a FIGO IIIB cervical cancer patient with extensive lymph node metastases in the pelvic and para-aortic areas. (A) External beam planning. A representative RT treatment plan includes the combination of an initial 4-field box technique (4500 cGy/25 frs) to the whole pelvis and para-aortic area with a subsequent IMRT boost with central pelvic sparing (900 cGy/5 frs) to the PET-detected lymph node basisn and parametria, and a final IGRT boost to the high SUV lymph nodes (720 cGy/4 frs). (B) PET-based intracavitary 3D brachytherapy planning to deliver at least 500 cGy to 90% of the PET-definied HR-CTV (as SUVs of 4.5 greater at the delayed phase) and IR-CTV (as SUVs of 2.5-4.5 at the delayed phase). The dark yellow arrow indicates a double-J in the right hydroureter. Ure, ureter; B, bladder; R, rectum; Si, sigmoid.
