# Supplementary material for: Patterns of failure after use of 18F-FDG PET/CT in integration of extended-field chemo-IMRT and 3D-brachytherapy plannings for advanced cervical cancers with extensive lymph node metastases
Source: BMC Cancer. 2016 Mar 3;16:179. doi: 10.1186/s12885-016-2226-0 (PMC4778334; doi:10.1186/s12885-016-2226-0)
Supplement: Additional file 5:Figure S5. — The integrated 18 F-FDG PET/CT staging, modern multi-modalities of radiotherapy (RT) planning and concurrent chemotherapy for treatment of an advanced cervical cancer patient with extensive pelvic, para-aortic and supraclavicular nodal diseases but no visceral metastasis at diagnosis. (A) An integrated concurrent chemoradiotherapy (CCRT) showing a combination of PET-guided cisplatin-based extended-field dose-escalating IMRT/IGRT and adaptive high-dose-rate (HDR) image-based intracavitary 3D brachytherapy. External beam RT (EBRT) and brachytherapy doses are transformed to EQD2 (equivalent dose of 2-Gy fraction) for combination. HR-CTV: SUVs of 4.5 greater at the delayed phase of PET; IR-CTV: SUVs of 2.5-4.5 at the delayed phase of PET; R, rectum; B, bladder; Si, sigmoid. (B) The hematologic toxicity profile of the patient during the extended-field CCRT. (DOC 258 kb) [file 12885_2016_2226_MOESM5_ESM.doc]

**Additional file 5: Fig. S5**

A

B


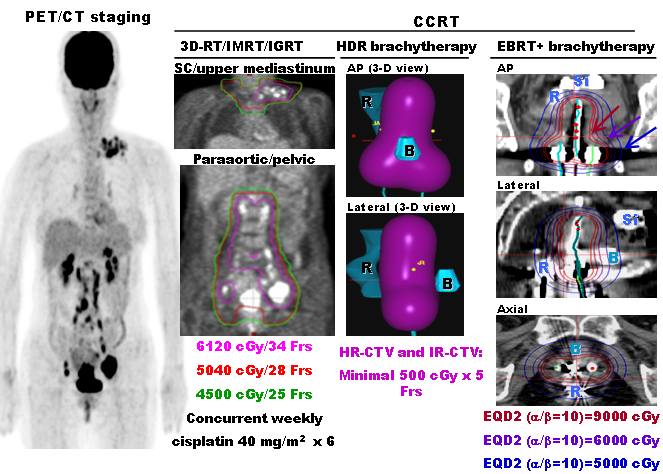


WBC (10^3^/μL)

HGB (g/dL)

Platelet(10^3^/μL)


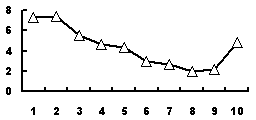


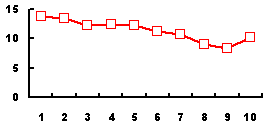


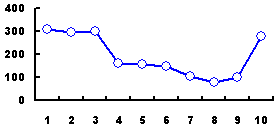


Weeks after Start of CCRT

Hematologic Profile

End of CCRT

Fig. S5. The integrated ^18^F-FDG PET/CT staging, modern multi-modalities of radiotherapy (RT) planning and concurrent chemotherapy for treatment of an advanced cervical cancer patient with extensive pelvic, para-aortic and supraclavicular nodal diseases but no visceral metastasis at diagnosis. (A) An integrated concurrent chemoradiotherapy (CCRT) showing a combination of PET-guided cisplatin-based extended-field dose-escalating IMRT/IGRT and adaptive high-dose-rate (HDR) image-based intracavitary 3D brachytherapy. External beam RT (EBRT) and brachytherapy doses are transformed to EQD2 (equivalent dose of 2-Gy fraction) for combination. HR-CTV: SUVs of 4.5 greater at the delayed phase of PET; IR-CTV: SUVs of 2.5-4.5 at the delayed phase of PET; R, rectum; B, bladder; Si, sigmoid. (B) The hematologic toxicity profile of the patient during the extended-field CCRT.
