# Supplementary material for: Patterns of failure after use of 18F-FDG PET/CT in integration of extended-field chemo-IMRT and 3D-brachytherapy plannings for advanced cervical cancers with extensive lymph node metastases
Source: BMC Cancer. 2016 Mar 3;16:179. doi: 10.1186/s12885-016-2226-0 (PMC4778334; doi:10.1186/s12885-016-2226-0)
Supplement: Additional file 6: Table S1. — Clinical characteristics and distribution of treatment modalities of 564 consecutive cervical cancer patients without visceral metastasis diagnosed in1990-2010. (DOC 38 kb) [file 12885_2016_2226_MOESM6_ESM.doc]

| Additional file 7: Table S1. Clinical characteristics and distribution of treatment modalities of 564 consecutive cervical cancer patients without visceral metastasis diagnosed in1990-2010. | | | |
| --- | --- | --- | --- |
| Years | 1990-2001 | 2002-2010 | Total |
| No. of Patients (%) | 229 | 335 | 564 |
| Age at diagnosis, years  Median  Range | 55.5  28.5-82.9 | 53.1  26.3-86.7 |  |
| Tumor histology  Squamous cell carcinoma  Adenocarcinoma  Adenosquamous  Others (small-cell, large-cell, clear-cell) | 175 (76.4)  18 (7.9)  29 (12.7)  7 (3.1) | 250 (74.6)  39 (11.6)  20 (6.0)  26 (7.8) | 425 (75.4)  57 (10.1)  49 (8.7)  33 (5.9) |
| FIGO clinical stage  IA2-IB2  IIA-IIB  IIIA-IIIB  IVA-IVB (paraaortic or supraclavicular lymph node metastasis but no distant organ metastasis)  Unknown | 90 (39.3)  80 (34.9)  35 (15.3)  19 (8.3)  5 (2.2) | 137 (40.9)  146 (43.6)  28 (8.4)  22 (6.6)  2 (0.6) | 227 (40.2)  226 (40.1)  63 (11.2)  41 (7.3)  7 (1.2) |
| Major treatment modality  Surgery ± adjuvant therapy  FIGO stage I  II  III  IV  Radiation ± chemotherapy  FIGO stage I  II  III  IV  Chemotherapy only  FIGO stage I  II  III  IV | 98 (42.8)  78 (34.1)  17 (7.4)  2 (0.9)  1 (0.4)  115 (50.2)  9 (3.9)  62 (27.1)  31 (13.5)  13 (5.7)  9 (3.9)  1 (0.4)  4 (1.7)  1 (0.4)  3 (1.3) | 152 (45.4)  122 (36.4)  28 (8.4)  1 (0.3)  1 (0.3)  172 (51.3)  14 (4.2)  114 (34.0)  27 (8.1)  17 (5.1)  4 (1.2)  0 (0)  1 (0.3)  0 (0)  3 (0.9) | 250 (44.3)  200 (35.5)  45 (8.0)  3 (0.5)  2 (0.4)  287 (50.9)  23 (4.1)  176 (31.2)  58 (10.3)  30 (5.3)  13 (2.3)  1 (0.2)  5 (0.9)  1 (0.2)  6 (1.1) |
| Curative treatment  Surgery alone  Surgery and radiation  Surgery and chemoradiotherapy  Surgery and chemotherapy  Radiation alone  Chemoradiotherapy | 58 (25.3)  21 (9.2)  16 (7.0)  5 (2.2)  61 (26.6)  51 (22.3) | 96 (28.7)  16 (4.8)  35 (10.4)  5 (1.5)  23 (6.9)  149 (44.5) | 154 (27.3)  37 (6.6)  51 (9.0)  10 (1.8)  84 (14.9)  200 (35.5) |
| FIGO, International Federation of Gynecology and Obsterics. | | | |
