# Supplementary material for: Patterns of failure after use of 18F-FDG PET/CT in integration of extended-field chemo-IMRT and 3D-brachytherapy plannings for advanced cervical cancers with extensive lymph node metastases
Source: BMC Cancer. 2016 Mar 3;16:179. doi: 10.1186/s12885-016-2226-0 (PMC4778334; doi:10.1186/s12885-016-2226-0)
Supplement: Additional file 7: Figure S6. — Clinical impacts of concurrent chemotherapy, IMRT and PET on survival of cervical cancer patients. The year 2002 represents a point at which our institution started to use 18 F-FDG PET/CT for staging, planning and/or follow-up in cancer patients, and commonly adopted the IMRT technique for cancer patients who needed RT. (A) Proportions of patients alive and free of disease at 8 years after treatment with a specific or combined treatment modality during the periods 1990–2001 and 2002–2010.The disease-free survival of patients treated by conventional RT in 1990–2001 was compared to that of patients treated by IMRT in 2002–2010 (conventional RT vs. IMRT). The disease-free survival of patients treated with concurrent chemoradiotherapy (CCRT) was compared to that of patients treated with RT alone (CCRT vs. RT alone). * represents p < 0.05 (Chi-squared test). S, surgery; RT, radiotherapy; C, chemotherapy; CCRT, concurrent chemoradiotherapy. (B) Kaplan-Meier survival estimates for invasive cervical cancer patients with or without PET or PET/CT for staging, planning, follow-up, and/or re-staging between 1990 and 2010. Although patients treated in 1990–2001 did not have pre-treatment PET staging, a portion of these patients had PET/CT for follow-up or re-staging when recurrence occurred after 2002. For patients treated in 2002–2010, if MRI did not show significant pelvic (cN1)/para-aortic (cM1) lymphadenopathy, parametrial involvement (cT2b), hydronephrosis (cT3b) and/or bladder/rectal invasion (cT4), no PET/CT was recommended according to our hospital guideline. For patients with recurrence noted after 2002, if PET/CT did not reveal distant metastasis, salvage surgery was performed if feasible. (DOC 51 kb) [file 12885_2016_2226_MOESM7_ESM.doc]

8-year Disease-free Survival of Invasive Cervical Cancer

A

**Additional file 6: Figure S6**

Alive and Free of Disease / Total Patients in Each Group (%)

*


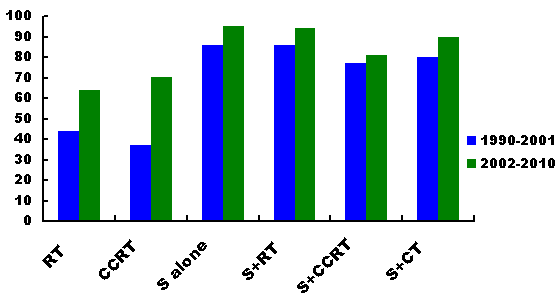


*

(Advanced FIGO stages)

Definitive Radiation Groups

(conventional pelvic RT 1990-2001)

(extended-field IMRT 2002-2010)

*

(Early FIGO stages)

Primary Surgery Groups +- adjuvant therapy

B

Impact of PET on cervical cancer patients 1990-2010


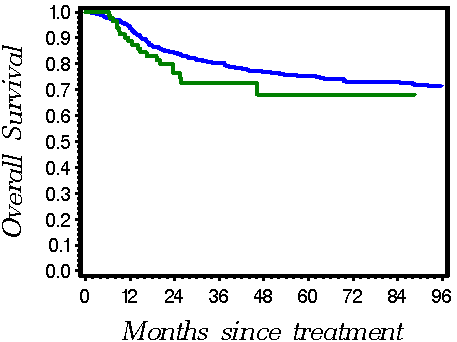


*p* = 0.1503

With PET (staging, RT planning, follow-up, and/or re-staging for salvage)

Without PET

Fig. S6. Clinical impacts of concurrent chemotherapy, IMRT and PET on survival of cervical cancer patients. The year 2002 represents a point at which our institution started to use ^18^F-FDG PET/CT for staging, planning and/or follow-up in cancer patients, and commonly adopted the IMRT technique for cancer patients who needed RT. (A) Proportions of patients alive and free of disease at 8 years after treatment with a specific or combined treatment modality during the periods 1990-2001 and 2002-2010.The disease-free survival of patients treated by conventional RT in 1990-2001 was compared to that of patients treated by IMRT in 2002-2010 (conventional RT vs. IMRT). The disease-free survival of patients treated with concurrent chemoradiotherapy (CCRT) was compared to that of patients treated with RT alone (CCRT vs. RT alone). ***** represents *p* < 0.05 (Chi-squared test). S, surgery; RT, radiotherapy; C, chemotherapy; CCRT, concurrent chemoradiotherapy. (B) Kaplan-Meier survival estimates for invasive cervical cancer patients with or without PET or PET/CT for staging, planning, follow-up, and/or re-staging between 1990 and 2010. Although patients treated in 1990-2001 did not have pre-treatment PET staging, a portion of these patients had PET/CT for follow-up or re-staging when recurrence occurred after 2002. For patients treated in 2002-2010, if MRI did not show significant pelvic (cN1)/para-aortic (cM1) lymphadenopathy, parametrial involvement (cT2b), hydronephrosis (cT3b) and/or bladder/rectal invasion (cT4), no PET/CT was recommended according to our hospital guideline. For patients with recurrence noted after 2002, if PET/CT did not reveal distant metastasis, salvage surgery was performed if feasible.
